# Crystal Structure Analysis Reveals Functional Flexibility in the Selenocysteine-Specific tRNA from Mouse

**DOI:** 10.1371/journal.pone.0020032

**Published:** 2011-05-24

**Authors:** Oleg M. Ganichkin, Ekaterina A. Anedchenko, Markus C. Wahl

**Affiliations:** Abteilung Strukturbiochemie, Freie Universität Berlin, Berlin, Germany; Victor Chang Cardiac Research Institute (VCCRI), Australia

## Abstract

**Background:**

Selenocysteine tRNAs (tRNA^Sec^) exhibit a number of unique identity elements that are recognized specifically by proteins of the selenocysteine biosynthetic pathways and decoding machineries. Presently, these identity elements and the mechanisms by which they are interpreted by tRNA^Sec^-interacting factors are incompletely understood.

**Methodology/Principal Findings:**

We applied rational mutagenesis to obtain well diffracting crystals of murine tRNA^Sec^. tRNA^Sec^ lacking the single-stranded 3′-acceptor end (^ΔGCCA^RNA^Sec^) yielded a crystal structure at 2.0 Å resolution. The global structure of ^ΔGCCA^RNA^Sec^ resembles the structure of human tRNA^Sec^ determined at 3.1 Å resolution. Structural comparisons revealed flexible regions in tRNA^Sec^ used for induced fit binding to selenophosphate synthetase. Water molecules located in the present structure were involved in the stabilization of two alternative conformations of the anticodon stem-loop. Modeling of a 2′-O-methylated ribose at position U34 of the anticodon loop as found in a sub-population of tRNA^Sec^ *in vivo* showed how this modification favors an anticodon loop conformation that is functional during decoding on the ribosome. Soaking of crystals in Mn^2+^-containing buffer revealed eight potential divalent metal ion binding sites but the located metal ions did not significantly stabilize specific structural features of tRNA^Sec^.

**Conclusions/Significance:**

We provide the most highly resolved structure of a tRNA^Sec^ molecule to date and assessed the influence of water molecules and metal ions on the molecule's conformation and dynamics. Our results suggest how conformational changes of tRNA^Sec^ support its interaction with proteins.

## Introduction

Certain bacteria, archaea and eukaryotes incorporate selenocysteine (Sec) into a fraction of their proteomes. These organisms require special biosynthetic pathways for the amino acid Sec and special decoding mechanisms for the co-translational insertion of Sec into selenoproteins on the ribosome. Specific tRNAs (tRNA^Sec^) are required for Sec biosynthesis and decoding, which are structurally distinct from canonical tRNAs.

Sec is the only amino acid that is exclusively synthesized on its cognate tRNA [1]. Initially, tRNA^Sec^ is aminoacylated with serine by seryl-tRNA synthetase (SerRS; [2]). In bacteria, Ser-tRNA^Sec^ is subsequently directly converted into Sec-tRNA^Sec^ by selenocysteine synthase (SelA), a pyridoxal phosphate-dependent enzyme that uses selenophosphate (SeP) as an activated selenium donor [3]. SeP is provided by selenophosphate synthetase (SelD) [4]. Archaea and eukaryotes require an additional activation step, during which *O*-phosphoseryl-tRNA^Sec^ kinase (PSTK) phosphorylates Ser-tRNA^Sec^ [5], [6], [7]. *O*-phosphoseryl-tRNA^Sec^ is then converted to Sec-tRNA^Sec^ by the archaeal/eukaryotic selenocysteine synthase (SecS; [8], [9], [10], [11]). Again, SeP serves as the selenium donor in this reaction and is provided by the archaeal/eukaryotic orthologs of selenophosphate synthetase, SPS in archaea and SPS2 in eukaryotes [9], [12].

For Sec insertion into selenoproteins, in-frame UGA codons, which would normally signal chain termination, are reprogrammed as Sec codons by a combination of *cis-* and *trans-*acting factors. Reprogramming requires selenocysteine insertion sequence (SECIS) elements in the selenoprotein mRNAs. In bacteria, SECIS elements are positioned directly downstream of the UGA Sec codon within the open reading frame [13]. In archaea and eukaryotes, SECIS elements are typically positioned in the 3′-untranslated regions [14]. SECIS elements are bound by the Sec-specific elongation factor SelB in bacteria [15] and by SECIS-binding protein 2 (SBP2) in eukaryotes [16]. Eukaryotic SBP2 links up with the eukaryotic functional homolog of SelB, eEFSec [17]. SelB and eEFSec also bind GTP and Sec-tRNA^Sec^ and deliver the latter molecule to the A site of the ribosome in response to a UGA Sec codon [18]. The archaeal Sec-specific elongation factor resembles its eukaryotic counterpart [19] but so far no archaeal adaptor protein equivalent to eukaryotic SBP2 has been discovered.

tRNA^Sec^ is the only component of the Sec synthesis and insertion pathways that participates throughout all steps of Sec biosynthesis and decoding. For this multi-tasking, it has to interact with a number of different proteins, *i.e.* SerRS, PSTK, SelA/SecS and SelB/eEFSec [20]. At the same time, tRNA^Sec^ may not interact with any of the other aminoacyl-tRNA synthetases or EF-Tu/eEF-1, in order to avoid erroneous incorporation of Sec at authentic UGA stop codons and in order to avoid incorporation of other amino acids at authentic Sec codons. The unique interaction profile of tRNA^Sec^ relies on special identity elements, which in part give rise to a number of distinct structural features but at the same time do not compromise the capacity of the molecule to act on the ribosome [21].

tRNA^Sec^ molecules are the longest tRNA species in cells, comprising 95 nucleotides (nts) in *Escherichia coli* [22] and 90 nts in mammals [21] compared with an average 75 nts in canonical tRNAs. Part of this additional length stems from the particularly long variable arms of tRNA^Sec^ molecules [21]. Furthermore, it has been suggested that the amino acid acceptor stem and the TΨC-stem of tRNA^Sec^, which coaxially stack in the 3D structure of tRNAs, together comprise 13 base pairs (bps) and are thus longer by one bp compared to the corresponding elements in canonical tRNAs [21], [22]. Canonical tRNAs exhibit seven bps in their acceptor stems and five bps in their TΨC-stems. For bacterial tRNA^Sec^, a model with eight bps in the acceptor stem and five base pairs in the TΨC-stem (8/5 model) has been confirmed based on structure probing in solution [22]. For eukaryotic tRNA^Sec^, mutually exclusive 7/5 [23] and 9/4 [21] models have been proposed. As yet another distinguishing feature, tRNA^Sec^ molecules were expected to encompass a six-bp dihydrouridine (D)-stem capped by a four-nt loop, compared to a four-bp D-stem and a seven to eleven-nt D-loop in canonical tRNAs. A recent crystal structure of human tRNA^Sec^ at 3.1 Å resolution verified the 9/4 model for eukaryotic tRNA^Sec^ and the unusual D-stem configuration [24]. Furthermore, that structure showed that eukaryotic tRNA^Sec^ lacks interactions between the D-stem and the variable arm, which results in a more open structure compared to canonical tRNAs. Finally, Sec-specific tRNAs are sparsely post-transcriptionally modified [25], resembling mitochondrial tRNA species in this regard [26].

We have used a rational approach to engineer mouse tRNA^Sec^ for crystallization and have determined its crystal structure at 2.0 Å resolution. While the general features of the molecule resemble the overall structure of human tRNA^Sec^, we discern significant global and local structural differences, including a flexible relative positioning of domains and two alternative conformations in the anticodon stem-loop. Comparison of our structure to the structure of human tRNA^Sec^ in complex with SecS [27] suggests that the RNA uses its flexibility to adapt to the protein partner. The two anticodon stem-loop conformations are differentially stabilized by water molecules and only one of the two forms is compatible with a specific nucleotide modification, known to occur in a subset of cellular tRNA^Sec^ in eukaryotes. Finally, we have delineated putative divalent metal ion binding sites by soaking with Mn^2+^, showing that unlike in many other RNAs, metal binding does not appear to be an important stabilizing principle in eukaryotic tRNA^Sec^.

## Results and Discussion

### RNA production and purification under non-denaturing conditions

We elaborated a quick and reliable scheme for the production and purification of crystallization-quality RNA samples, which combines optimized *in vitro* transcription on PCR-generated templates with two chromatographic purification steps, avoiding any denaturing step or phenol/chloroform extraction. The approach can be efficiently combined with combinatorial mutagenesis to generate a collection of RNA constructs for crystallization.

**Figure 1:**
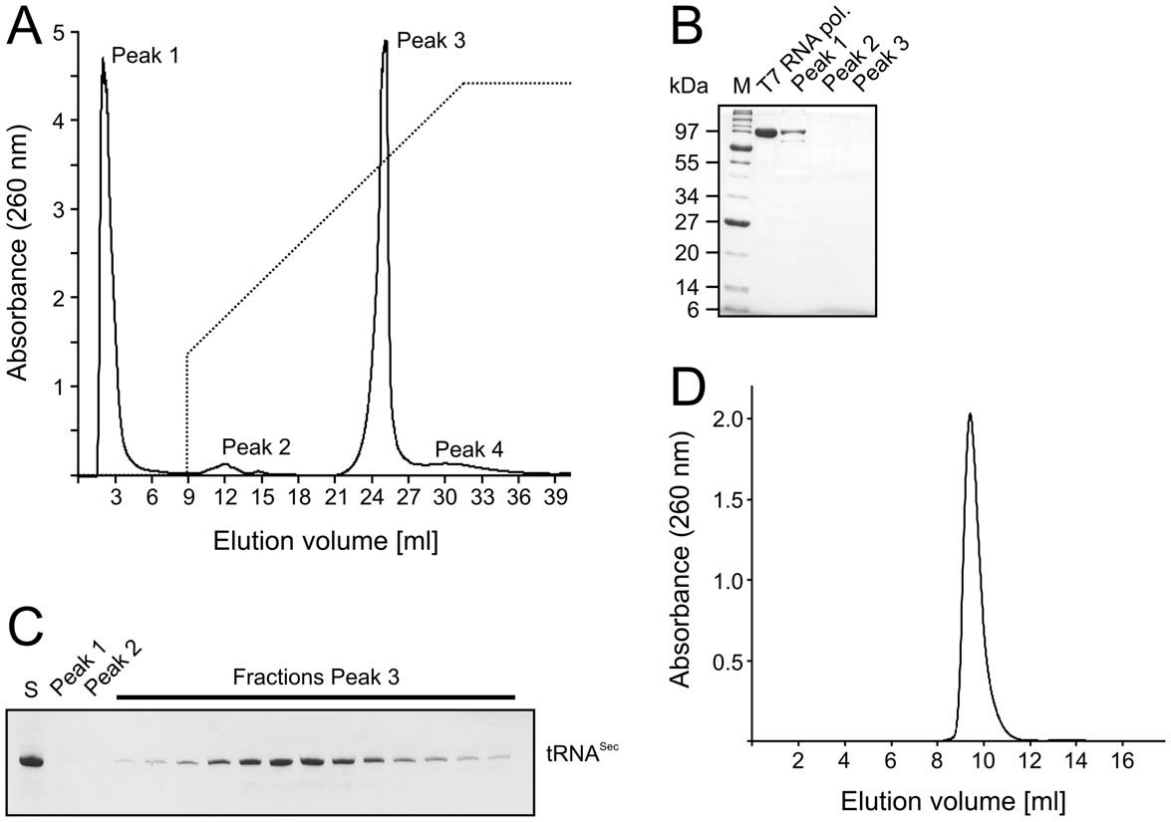
Non-denaturing RNA purification. (**A**) Elution profile of *in vitro* transcribed mouse tRNA^Sec^ from a MonoQ column. Peak 1 – unincorporated rNTPs, T7 RNA polymerase and other proteins; Peak 2 – abortive synthesis transcripts; Peak 3 – desired RNA sample; Peak 4 – aggregates or higher molecular weight nucleic acids. The gradient (buffer B from 30 to 100%) is shown as a dashed line. (**B**) Denaturing SDS PAGE analysis of peak fractions from Peaks 1–3. T7 RNA polymerase and molecular weight markers (M) were loaded as references. Protein bands were stained with Coomassie. (**C**) Denaturing urea PAGE analysis of peak fractions eluted from the MonoQ column. S – crude transcription extract. RNA bands were stained with methylene blue. (**D**) Elution profile of mouse tRNA^Sec^ from a Superdex 75 10/300 GL column.

We PCR-assembled DNA fragments encompassing a T7 RNA polymerase promoter and the RNA coding region and inserted the constructs into plasmids. The plasmids served as templates for amplification of the promoter and insert regions, using reverse primers with two 2′-*O*-methyl modified nucleotides at their 5′-ends to avoid 3′-end heterogeneity of the RNA products in the subsequent transcription reactions [28], [29]. The modified PCR products served as templates for *in vitro* transcription by T7 RNA polymerase. After removal of the DNA template by incubation with RNase-free DNase, the sample was directly loaded on a strong anion exchange column. None of the proteins present in the reaction mixture bound to the column under conditions, which afforded efficient binding and separation of RNAs (Figures 1A–C). Subsequently, size exclusion chromatography was used to remove aggregates or misfolded species with different hydrodynamic volumes and to put the RNA samples into the crystallization buffer. The target RNAs typically eluted in a single sharp peak (Figure 1D). We deliberately avoided a concentrating step *via* ethanol or isopropanol precipitation, which can re-introduce aggregation. *Via* the outlined protocol, we obtained around 3.0 mg of tRNA-sized RNAs in pure form from 2 ml transcription reactions.

**Figure 2:**
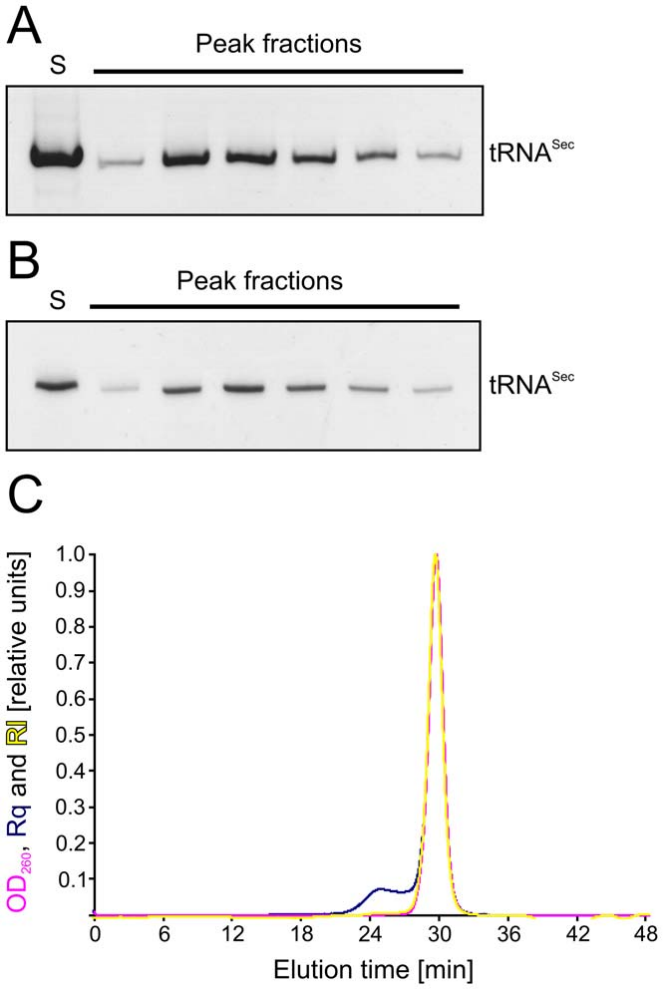
Analysis of purified RNA. (**A**) Native and (**B**) denaturing PAGE analysis of fractions collected from Superdex 75 10/300 GL gel filtration. S – concentrated RNA sample after anion exchange chromatography. (**C**) Analysis of mouse tRNA^Sec^ by multi-angle static light scattering. The optical density at 260 nm (OD_260_; magenta), Rayleigh ratio (Rq; blue) and differential refractive index (RI; yellow) were monitored during analytical gel filtration on a Superdex 200 10/300 GL column. The measurement was done by Wyatt Technology Europe GmbH.

The above two-step procedure yielded RNAs that were both chemically pure and conformationally homogeneous as seen from analytical denaturing and non-denaturing gel electrophoresis, respectively (Figures 2 A,B). In the case of a mouse tRNA^Sec^ variant lacking a single-stranded 3′-overhang (^ΔGCCA^tRNA^Sec^), the sample was also analyzed by multi-angle static light scattering, confirming that it was largely monomeric and monodisperse (Figure 2C).

### Design of tRNA^Sec^ variants

**Figure 3:**
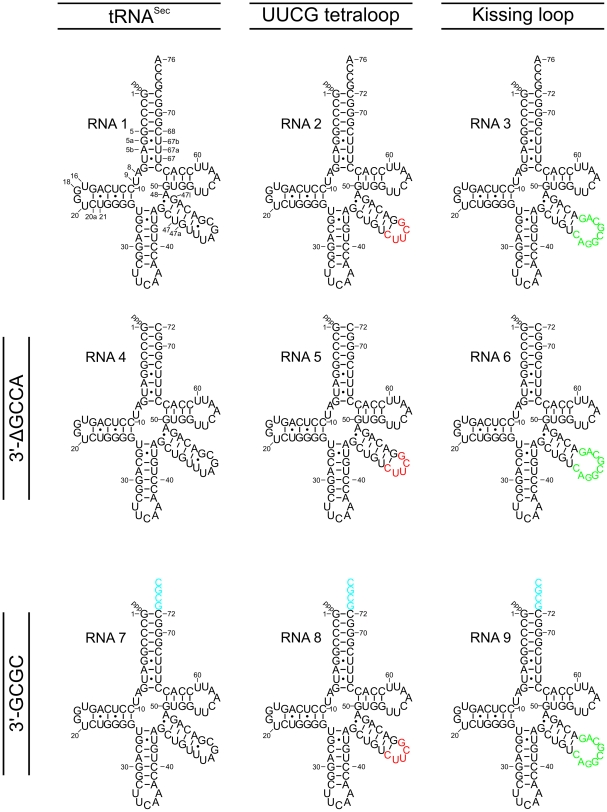
tRNA^Sec^ constructs for crystallization screening. RNA 1 represents full-length tRNA^Sec^. Canonical tRNA numbering was used throughout. Additional nucleotides (labeled with lower case Latin characters) and gaps (missing numbers) compared to the canonical tRNA numbering are indicated only in the scheme of RNA 1. RNAs 2 and 3 were created by site-directed mutagenesis and contain a UUCG (red) or a kissing loop (green) in place of the wt variable loop, respectively. Using the initial, mutated constructs, further DNA templates were generated for *in vitro* transcription, which allowed synthesis of tRNA^Sec^ species with deletion of the 3′-GCCA end (RNAs 4, 5 and 6) or with substitution of the 3′-GCCA with a self-complementary 3′-GCGC overhang (RNAs 7, 8 and 9).

Purified mouse tRNA^Sec^ crystallized readily under many different crystallization conditions but crystals exhibited low diffraction quality. In order to obtain better diffracting crystal forms, we generated mutant tRNA^Sec^ molecules, which exhibited novel crystal packing potentials or contained elements that were expected to increase their conformational stabilities (Figure 3). We attempted substitution of the variable loop with a kissing loop [30] (to allow dimer formation) or with a UUCG tetraloop (to increase thermodynamic stability), introduction of a self-complementary 3′-overhang at the acceptor arm (to allow dimerization) and deletion of the 3′-GCCA overhang (to enhance stacking capacity at this end of the molecule). All constructs were predicted to retain the key structural features of the wild type (wt) molecule.

To minimize cloning steps during the synthesis of the entire collection of RNAs, we first used site-directed mutagenesis to introduce sequence substitutions in the variable loop, based on which we subsequently generated by PCR nine different templates (Figure 3). Since our strategy was based on PCR-amplified DNA templates for *in vitro* transcription, we could efficiently parallelize the production of the RNA constructs. The same purification conditions could be employed for wt and mutant tRNAs so that no additional optimization of purification parameters was required.

### Crystallization and structure solution

Stacking *via* terminal bps is an important packing principle found in numerous RNA and RNA complex crystal structures. Thus, as one strategy for crystallization of tRNA^Sec^, we deliberately deleted the single stranded 3′-GCCA overhang, generating ^ΔGCCA^tRNA^Sec^ with a blunt-ended acceptor stem. ^ΔGCCA^tRNA^Sec^ crystallized in space group P2_1_ and yielded a diffraction data set to 2.0 Å resolution. In the following, we refer to the ^ΔGCCA^tRNA^Sec^ construct as “tRNA^Sec^” for simplicity.

**Figure 4:**
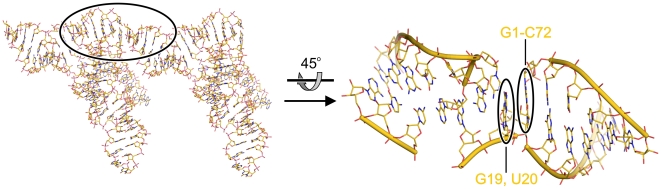
Crystal contacts of ^ΔGCCA^tRNA^Sec^. Stacking interactions of the terminal G1-C72 bp of the acceptor stem of molecule A with nucleotides G19 and U20 of the the D-loop of a neighboring molecule A. Left – overview. Right – close-up of the crystal packing interaction. In this and the following figures, atoms are color-coded in identical fashion; carbon – gold (or as the respective molecule); nitrogen – blue; oxygen – red; phosphorus – orange. Hydrogen bonds are indicated by dashed lines. The right view is rotated 45° about the horizontal axis as indicated.

The structure of mouse tRNA^Sec^ was solved by molecular replacement using portions of human tRNA^Sec^ ([24]; PDB ID 3A3A) as search models and refined including all data to 2.0 Å resolution with good stereochemistry (Table 1). There were two molecules of tRNA^Sec^ in an asymmetric unit of the present crystal form (referred to as molecules A and B). All 86 nucleotides of both ^ΔGCCA^tRNA^Sec^ molecules could be clearly located in the electron density map. Notably, the blunt-ended acceptor stem mediated crucial crystal packing contacts as intended (Figure 4). The terminal G1-C72 bp of molecule A stacked on residues G19 and U20 of the D-loop of a symmetry-related molecule A (Figure 4). Similarly, the terminal G1-C72 bp of molecule B stacked on the D-loop of a symmetry-related molecule B. Thus, deletion of the 3′-overhangs may be a general strategy to obtain diffracting crystals also of other tRNAs as well as of tRNA complexes, for which these overhangs are not required. This idea is supported by the observation that some tRNAs even use stacking *via* the acceptor stem for crystal packing with the single-stranded 3′-end moved out of the way or degraded [31].

**Table 1:** Crystallographic data and refinement statistics.

Data Collection	Native	Manganese
**Wavelength** (Å)	0.91841	1.89218
**Temperature** (K)	100	100
**Space group**	P2_1_	P2_1_
**Unit cell parameters**		
a, b, c (Å)	48.9, 96.1, 71.8	48.4, 96.1, 71.8
ß (°)	104.8	104.9
**Resolution** (Å)	50.00-2.00 (2.03-2.00)^a^	35-2.84 (2.91-2.84)
**Reflections**		
Unique	42189 (2072)	28639 (2067)
Completeness (%)	97.9 (95.8)	97.1 (95.6)
Redundancy	3.9 (3.7)	1.9 (1.8)
I/σ(I)	19.7 (1.2)	12.4 (2.6)
R_sym_(I)^b^	4.7 (80.7)	5.3 (31.8)
**Refinement**		
**Resolution** (Å)	50.00-2.00 (2.05-2.00)	
**Reflections**		
Number	40040 (2700)	
Completeness (%)	97.4 (90.1)	
Test set (%)	5.0	
**R_work_** c	21.7 (37.6)	
**R_free_** c	25.2 (42.3)	
**ESU** (Å)^d^	0.134	
**Contents of A.U.** e		
RNA molecules/residues/atoms	2/172/3888	
Water oxygens	195	
SO_4_^2−^	2	
Acetate	2	
Glycerol	2	
**Mean B-factors** (Å^2^)		
Wilson	46.8	
RNA	46.9	
Water	43.5	
Solutes	48.6	
**Rmsd from target geometry**		
Bond Lengths (Å)	0.010	
Bond Angles (°)	1.748	
**Rmsd B-factors** (Å^2^)		
Main chain bonds	0.389	
Main chain angles	0.647	
Side chain bonds	1.597	
Side chain angles	2.330	
**PDB ID**	3RG5	

a Data for the highest resolution shell in parentheses.

b R_sym_(I) = Σ_hkl_Σ_i_|I_i_(hkl)−<I(hkl)>|/Σ_hkl_Σ_i_|I_i_(hkl)|; for n independent reflections and i observations of a given reflection; <I(hkl)> – average intensity of the i observations.

c R = Σ_hkl_||F_obs_|−|F_calc_||/Σ_hkl_|F_obs_|; R_work_−hkl∉T; R_free_−hkl ∈ T; R_all_ – all reflections; T – test set.

d ESU – estimated overall coordinate error based on maximum likelihood.

e A.U. – asymmetric unit.

### Overall structure of tRNA^Sec^

**Figure 5:**
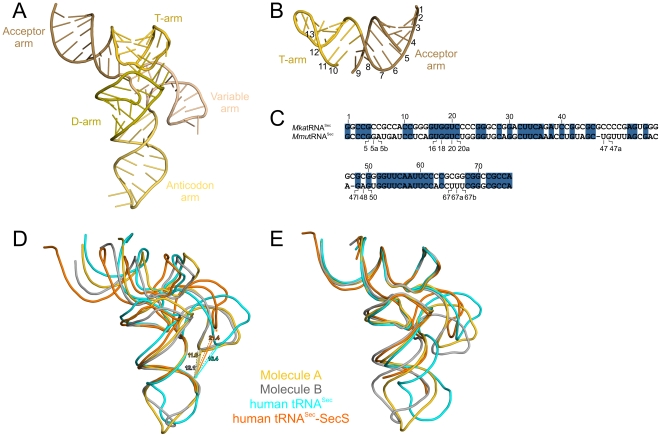
Structure of mouse tRNA^Sec^ compared to human tRNA^Sec^. (**A**) Overall 3D structure of mouse tRNA^Sec^ with structural elements highlighted by colors. (**B**) A 13 bp helix formed by coaxial stacking of the acceptor and T-stems. Numbers refer to the base pairs. (**C**) Sequence alignment of tRNA^Sec^ from *Methanopyrus kandleri* (*Mka*) and *Mus musculus* (*Mmu*) performed with ClustalW2 (http://www.ebi.ac.uk/Tools/clustalw2/index.html). Additional nucleotides (labeled with lower case Latin characters) and gaps (missing numbers) compared to the canonical tRNA numbering are indicated below the alignment. (**D**) Ribbon models of mouse tRNA^Sec^ (this work; molecule A and molecule B colored gold and silver respectively) superimposed on human tRNA^Sec^ structures based on phosphorus atoms in the anticodon stems (derived from PDB entries 3A3A [isolated human tRNA^Sec^; cyan] and 3HL2 [human tRNA^Sec^ in complex with SecS; orange]). Distances between phosphorus atoms of G47h (variable arm) and G30 (anticodon arm) are shown with arrows. (**E**) Superposition of the same molecules based on phosphorus atoms of the acceptor and T-arms.

The two crystallographically independent tRNA^Sec^ molecules globally resemble each other (root-mean-square deviation [rmsd] of 1.6 Å for 77 phosphorus atoms) but exhibit differences in detail (see below). The global 3D structure of tRNA^Sec^ conforms to the canonical L-shape of tRNAs (Figure 5A). As predicted [21] and as recently seen in the crystal structure of human tRNA^Sec^ [24], tRNA^Sec^ contains a number of unique architectural features.

#### Anticodon and TΨC-arms

Based on the secondary structure models of tRNA^Sec^ and on structure probing in solution, a 13-bp composite acceptor-TΨC helix was predicted and proposed as a critical distinguishing feature to the twelve-bp stack that these elements form in canonical tRNAs [21], [22]. However, biochemical analyses indicated a possible difference in the number of bps for the two stems in the different phylogenetic kingdoms. For bacterial tRNA^Sec^, structure probing had suggested an eight-bp anticodon stem and a five-bp TΨC-stem [21], [22]. For eukaryotic tRNA^Sec^, two different studies had suggested two mutually exclusive 7/5 [23] or 9/4 [21] distributions.

In agreement with the structure of human tRNA^Sec^ [24], we clearly see nine-bp anticodon stems and four-bp TΨC-stems in both crystallographically independent mouse tRNA^Sec^ molecules, showing unequivocally that the 9/4 structure is the functionally relevant conformation (Figure 5B). The structure of human tRNA^Sec^ in complex with SecS has recently revealed the functional importance of the 13 bp acceptor-TΨC helix for Sec biosynthesis [27]: SecS forms a tetramer with one dimer serving as the predominant binding platform for PSer-tRNA^Sec^ *via* the composite acceptor-TΨC helix and the longer acceptor-TΨC helix is required for the 3′-end to reach into the active site of the neighboring catalytic dimer of SecS.

#### Dihydrouridine arm

The D-arm of tRNA^Sec^ was predicted to comprise a six-bp stem capped by a four-nt loop, compared with the four-bp D-stem and the variable seven to ten-nt D-loop in canonical tRNAs. This prediction is borne out by our structure of mouse tRNA^Sec^ (Figure 5A) and the same organization was also found in human tRNA^Sec^ [24]. The length of the D-stem serves as an identity element for PSTK, as shown in the recent co-crystal structures of an archaeal tRNA^Sec^ [32] (PDB ID 3ADB) and of an engineered tRNA^Sec^ [33] (PDB ID 3AM1) with the enzyme. The importance of the long D-stem as a recognition element for PSTK was further underscored by the observation that in some archaeal tRNA^Sec^ species it is further elongated to seven bps [33].

The sequence of archaeal tRNA^Sec^ species is only ca. 55% identical to mouse tRNA^Sec^ (Figure 5C). Their structures deviate significantly from the structure of mouse tRNA^Sec^ (rmsd between 2.2 and 3.0 Å for 56 to 61 common phosphorus atoms for different pairs of molecules) primarily due to local conformational differences in the AD-linkers (see below), the acceptor and the anticodon arms. However, due to the large sequence divergence between mouse and archaeal tRNA^Sec^ molecules, these conformational differences cannot be directly related to induced fit rearrangements upon PSTK binding.

#### Variable arm and AD-linker

**Figure 6:**
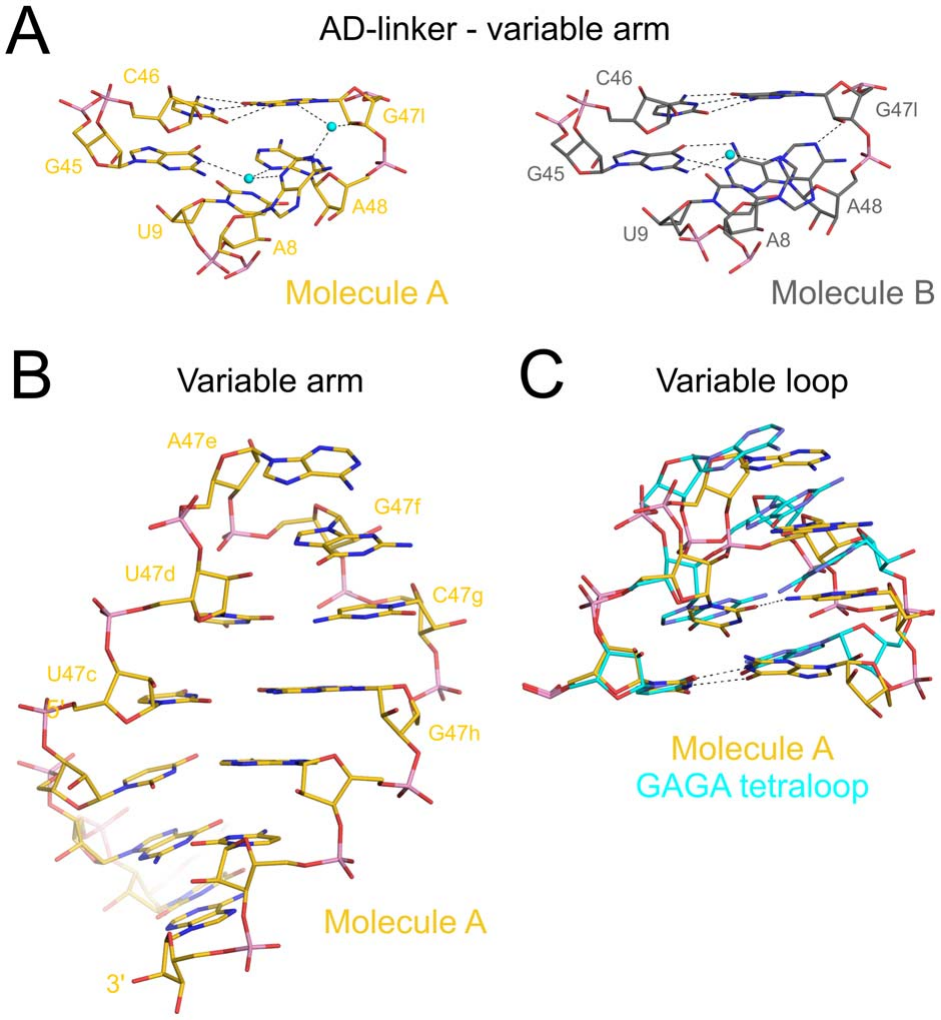
Tertiary interactions of the AD-linker and variable arm. (**A**) Water-mediated interaction of the AD-linker with the first bp of the variable arm in mouse tRNA^Sec^ (molecule A – gold; molecule B – silver). Water molecules – cyan spheres. Hydrogen bonds are shown as dashed lines. (**B**) Close-up of the variable arm (molecule A). Nucleotides of the variable loop are labeled. (**C**) Superposition of the mouse tRNA^Sec^ variable loop (carbon – gold) with a stable GAGA tetraloop from the 23S rRNA sarcin/ricin domain (carbon – cyan; PDB ID 483D).

The exceptionally long variable arm of mouse tRNA^Sec^ comprises a six-bp stem and a four-nucleotide loop and protrudes from one side of the molecule (Figure 5A). The first and last nucleotides of the variable arm (G45 and A48) form a water-mediated contact in one of the tRNA^Sec^ molecules of the asymmetric unit of the present crystal structure (molecule A) and a direct non-canonical purine-purine pair in the other (molecule B; Figure 6A). Irrespective of whether the non-canonical G45•A48 bp is formed, A48 efficiently stacks on U9 of the region connecting the anticodon and D-arms (AD-linker comprising A8 and U9; Figure 6A). In contrast, A8 of the AD-linker is positioned variably in molecules A and B and is connected *via* water molecules to the first and last nts of the variable stem (Figure 6A). The molecule B conformation including the non-canonical G45•A48 bp was also observed in the structure of human tRNA^Sec^ and in the complex of tRNA^Sec^ with SecS [27]. It remains to be seen whether the alternative conformation with a broken G45•A48 bp and a differently positioned A8 as revealed in molecule A may be important for binding of tRNA^Sec^ by other factors.

Beyond the above interactions with the AD-linker, the variable arm does not engage in contacts with the D-stem, as has also been observed for human tRNA^Sec^ [24]. As a consequence, tRNA^Sec^ lacks a closely packed core in the area where the five helices of the structure meet as seen in canonical tRNAs.

The loop of the variable arm of mouse tRNA^Sec^ includes bases 47d to 47g (UAGC). This loop is sealed by a U47c•G47h wobble pair (Figure 6B). U47d and C47g also form a non-canonical bp with a single hydrogen bond between O2 of U47d and N4 of C47g. Possibly there is also a weak CH—O interaction [34], [35] between O2 of U47d and C5H of C47g. However, there are no bridging water molecules as often seen between pyrimidine-pyrimidine base pairs in RNA duplexes [36]. As a consequence of the latter base pair, the loop of the variable arm comprises only two unpaired nucleotides. The bases of A47e, G47f and C47g form a continuous stack on the 3′-side of the loop with their Watson-Crick functional groups turned outwards. This organization allows G47f and C47g to mediate crystal contacts *via* their Watson-Crick faces with symmetry-related C47g and G47f nts, respectively. The first loop nucleotide, U47d, is connected *via* a sharp kink in the backbone to A47e, forming a U-turn motif [37].

The non-canonical base pair between the first and last nts of the loop, the three-nt stack on the 3′-side of the loop and the U-turn are known features also found in thermodynamically stable UUCG or GNRA tetraloops [38], [39]. To investigate whether the loop of the tRNA^Sec^ variable arm conforms to a related motif, we superimposed the present structure on the structure of the GAGA tetraloop derived from the 23S rRNA sarcin/ricin loop ([40]; Figure 6C). While the loop of the variable arm resembles the 3D structure of GNRA tetraloops, detailed inspection revealed that the distance between N3 of U47d and the 2′-OH group of C47g is too long for a hydrogen bond (3.9 Å in molecule A, 4.3 Å in molecule B). In addition, the non-canonical nucleotide pairs within GNRA and UUCG tetraloops comprise two direct hydrogen bonds (between the bases or between base and backbone). Since these features are critical determinants for the stability of GNRA and UUCG tetraloops [41], we conclude that the loop of the variable arm is not equally stabilized.

### Functional flexibility in tRNA^Sec^

Mouse tRNA^Sec^ differs in only one position in the variable arm from human tRNA^Sec^ (U47c vs. C47c), whereby the variable loop-closing C-G bp of human tRNA^Sec^ is replaced by a U•G wobble pair. Thus, not surprisingly, the mouse tRNA^Sec^ structures are globally similar to the overall structure of human tRNA^Sec^ (rmsd of 1.1 Å for 63 phosphorus atoms, molecule A and rmsd of 2.1 for 68 phosphorus atoms, molecule B). However, we discerned a number of conformational differences between the molecules on the local scale. Since the sequences are almost identical, the structural differences we see between mouse and human tRNA^Sec^ complement the picture of the flexibility within the molecule, which emerges from comparison of our crystallographically independent copies of mouse tRNA^Sec^ alone (see above). Together, the different conformations directly reflect part of the structural repertoire available to tRNA^Sec^, which will be important for the biological functions of the molecule.

#### Flexible relative domain orientation

Comparison of the crystallographically independent mouse tRNA^Sec^ structures and their comparison to the structures of human tRNA^Sec^ alone [24] or in complex with SecS [27] showed that the variable and anticodon arms can adopt different relative orientation with respect to each other and with respect to the remainder of the molecule (Figures 5D,E). In the present crystal structure, the variable arm is positioned closest to the anticodon arm (Figure 5D). In the structures of human tRNA^Sec^ the variable arm is positioned further away from the anticodon arm with the largest opening seen in the co-crystal structure with SecS. We do not see direct contacts between the variable and anticodon arms in our structure and there are no water-mediated bridges between these two elements, suggesting that the structural differences among the isolated tRNA^Sec^ structures are perhaps induced by different crystal packing. The malleability of the variable arm may stem from the absence of tertiary interactions between the variable and the D-arm as described above.

Irrespective of the source, the analysis shows that the flexible variable arm can undergo induced fit adjustment during interaction of tRNA^Sec^ with proteins. The structural flexibility of tRNA^Sec^, exemplified by the variable arm, most likely provides an indirect recognition mechanism. In agreement with this notion, flexible regions in other tRNAs have previously been shown to be important for recognition by various enzymes involved in translation or tRNA modification [42].

#### Alternative conformations in the anticodon arm

The anticodon loop of tRNA^Sec^ in complex with SecS was disordered [27] but all structures of isolated tRNA^Sec^ molecules, including both crystallographically independent molecules of the present structure, showed clear electron density for this region. Nevertheless, alignment of the available isolated tRNA^Sec^ structures is consistent with the picture that emerged from the tRNA^Sec^-SecS complex, supporting a considerable flexibility of the anticodon loop.

**Figure 7:**
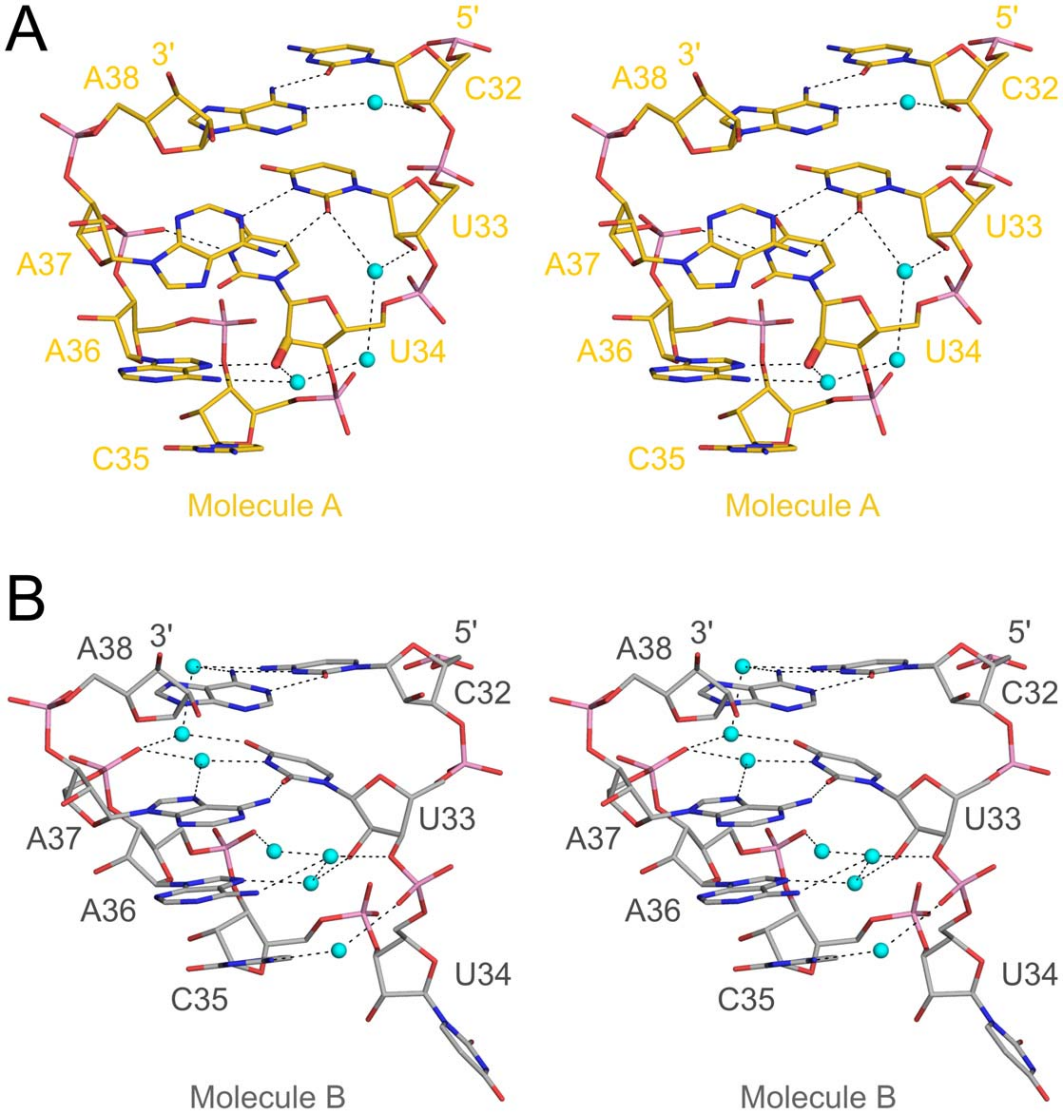
Anticodon loop conformations in mouse tRNA^Sec^. Stereo stick model of the closed anticodon loop conformation of molecule A (**A**) and of the open anticodon loop conformation of molecule B (**B**). The 2′-oxygen of the U34 ribose in molecule A that is methylated in a subset of cellular tRNA^Sec^ is shown as a thicker stick. Water molecules – cyan spheres. Hydrogen bonds are indicated by dashed lines.

The anticodon loops of the two tRNA^Sec^ molecules of the present structure adopt two distinct conformations (Figures 7A,B) that both differ significantly from the conformation seen in isolated human tRNA^Sec^ [24]. In the latter molecule, the anticodon loop adopts an irregular structure due to extensive crystal packing interactions. In molecule A of the present structure, the anticodon stem consists of eight bps with a three-nt loop, comprising the three anticodon bases, U34, C35 and A36. The bases of C35 and A36 stack on each other with their Watson-Crick functional groups turned outwards. However, U34 forms a U-turn motif whereby the base is “tugged away” below a U33•A37 reverse Watson-Crick base pair that closes the loop (Figure 7A). As a consequence, the N3 atom of U34 forms a hydrogen bond to a non-bridging phosphate oxygen of A37 so that its Watson-Crick face is not available for base pairing with the UGA Sec codon in the mRNA. U34 also partially wedges between A36 and A37 at the 3′-end of the loop, interfering with full stacking between the bases of these nts (Figure 7A). Taken together, the anticodon loop conformation of molecule A clearly is non-functional for codon recognition on the ribosome.

The overall structure of the anticodon loop in molecule B closely resembles the classical anticodon loop of tRNA^Phe^ [43]. In the anticodon loop of molecule B the reverse Watson-Crick bp between U33 and A37 is replaced by a partially opened reverse Hoogsteen pair, which comprises a single direct hydrogen bond between O2 of U33 and N6 of A37 and a water-mediated contact between N3 of U33 and N7 of A37 (Figure 7B). U34 is now completely flipped outwards and does not interact with any other nucleotide in the loop. After the removal of U34 from the inside of the loop, the repositioned A37 fully stacks with A36, which in turn maintains its stacking with C35 from the anticodon. The position of U34 at the base of the loop seen in the first conformation is occupied by U33 in the second conformation, allowing it to maintain additional water-mediated contacts to the phosphate of A37. Open anticodon arm conformations with five-nt loops and seven bp stems are favorable for binding of the tRNAs to the A-site of the ribosome [44], [45]. We therefore suggest that the conformation of the anticodon arm seen in molecule B reflects the functional conformation for UGA-decoding on the ribosome.

#### The functional anticodon loop conformation of tRNA^Sec^ is favored by 2′-O-methylation of U34

tRNA^Sec^ studied herein was produced by *in vitro* transcription and thus lacked post-transcriptional modifications. Certain modifications can have pronounced effects on the conformation of the anticodon loop, stabilizing a conformation that is capable of recognizing the cognate codons on mRNAs. Comparative NMR studies of A37-N^6^-dimethylallyl-modified and unmodified anticodon stem-loops of *E. coli* tRNA^Phe^ revealed that in the absence of the modification, a closed, three-nt loop conformation was preferentially adopted at the expense of the (functional) open, five-nt loop conformation [37]. Moreover, computational analysis of tRNA^Cys^ showed that another modification at position 37 (ms^2^i^6^A) is important for the disruption of a base pair, which could form between unmodified A37 and A38 with U32 and U33 [42].

Although tRNA^Sec^ is generally sparsely modified [46], two differentially modified pools of tRNA^Sec^ exist in eukaryotic cells. The first population contains 5-methylcarboxymethyl-uridine (mcm5U) at position 34 and N6-isopentyl-adenosine (i^6^A) at position 37 [25]. The second population exhibits in addition a 2′-O-methylated ribose (mcm5Um) at position 34. Using our present crystal structure, we tried to assess if and how the differential 2′-O-methylation of U34 may affect the anticodon loop conformation of tRNA^Sec^. Notably, a 2′-O-methyl ribose at U34 will destabilize the closed, presumed inactive anticodon loop conformation seen in molecule A (Figure 7A). In that structure, the modification would abolish formation of two contacts involving the unmodified U34, between the U34 2′-hydroxyl group and N7 of A37 and a water molecule between O2′ of U34 and N6 of A37. We therefore suggest that ribose methylation of U34 will lead to preferential adoption of the open, presumed active five-nt loop conformation. In general agreement with this conclusion, structure probing experiments have shown that tRNA^Sec^ bearing a (mcm5U34m)-C35-A36 anticodon loop adopted a more open conformation, while a more compact conformation was seen in tRNA^Sec^ bearing a (mcm5U34)-C35-A36 anticodon [46]. While also the presence of a modified i^6^A at position 37 is important for efficient decoding of UGA Sec codons by tRNA^Sec^ [47], i^6^A37 appears to be compatible with both conformations seen in our crystal structure and we thus cannot explain the role of this modification at the present time.

### Hydration of tRNA^Sec^

**Figure 8:**
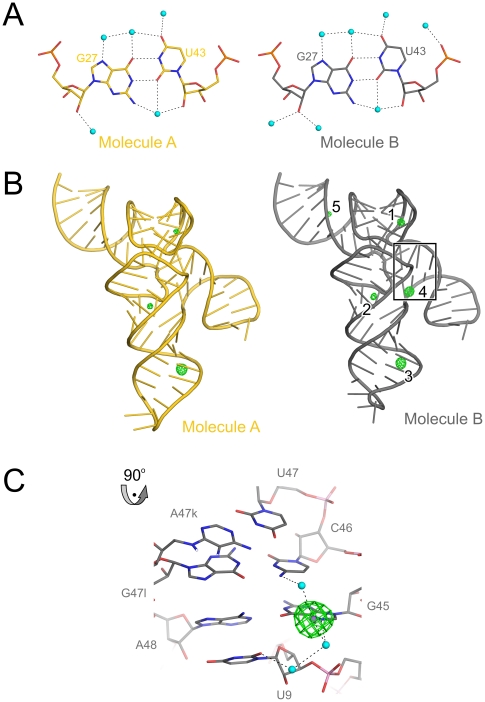
Hydration and metal ion binding of mouse tRNA^Sec^. (**A**) Hydration of the G27•U43 wobble bps in molecules A (carbon – gold) and B (carbon – silver) of mouse tRNA^Sec^, showing a full complement of first-shell water molecules (cyan spheres). Hydrogen bonds are indicated by dashed lines. (**B**) Anomalous difference Fourier map contoured at the 5 σ level (green mesh) calculated with anomalous differences recorded from a Mn^2+^-soaked crystal and phases obtained from molecular replacement with the native structure as a search model. Molecule A – gold; molecule B – silver. There are three common Mn^2+^ sites (1–3) in the two tRNA^Sec^ molecules. Sites 4 and 5 were found only in chain B. The boxed region is shown in a close-up view in the following panel. (**C**) Close-up of the boxed region of panel (B). Mn^2+^ ion (site 4; purple sphere) apparently reinforcing the interaction of U9 (AD-linker) with A48•C45 (first bp of the variable arm).

Water molecules are intimately involved in the folding of nucleic acids and in mediating functions such as molecular recognition or catalysis by these molecules [48], [49]. Indeed water can be considered an integral part of nucleic acid structures [50], [51]. Previous human tRNA^Sec^ structures were determined at 2.8 to 3.1 Å resolution, where only very few strongly ordered water molecules could be seen. We located 195 water molecules distributed around the two molecules of mouse tRNA^Sec^ in the present crystal structure. As seen previously in other RNA structures [52], the 2′-hydroxyl groups of the riboses are heavily hydrated (more than 25% of all hydrogen bonds involving water molecules) in a manner that will effectively stabilize the overall structure by “cross-strutting”. 2′-hydroxyl groups are water-bridged primarily to (1) functional groups of the bases (in particular to guanine N3 and cytosine O2 atoms), (2) to the 2′-hydroxyl groups of neighboring nucleotides, (3) to neighboring O4′ atoms and (4) to neighboring phosphate moieties. An illustrative example is afforded by the hydration “webs” seen around G•U base pairs (Figure 8A). The same characteristic hydration pattern has been observed earlier in structures of RNA duplexes [53], [54]. Water molecules are also intimately involved in stabilizing alternative structures, including the presumed functional conformation of the anticodon loop of molecule B (Figure 7B).

### Metal ion binding sites

Many RNA molecules are functionally stabilized by divalent metal ions, predominantly by Mg^2+^. Although G•U pairs form potential divalent metal ion binding sites in RNA [55] and although the crystallization buffer contained 10 mM Mg^2+^, we failed to discern any bound Mg^2+^ ions either in the vicinity of the G•U pairs or elsewhere in the molecules. In order to assign potential weak divalent metal ion binding sites, we soaked a crystal in high concentrations (100 mM) of Mn^2+^, a known surrogate for Mg^2+^ [56], [57], and collected a diffraction dataset at long X-ray wavelength, where Mn^2+^ exhibits an anomalous signal (Table 1). Indeed, eight Mn^2+^ sites were found in an anomalous difference Fourier map (Figure 8B). Three metal ions were associated with molecule A and five metal ions bound at molecule B (Figure 8B). All metal ions interacted with an N7 position of a guanine residue. At the lower resolution of the derivative dataset (2.8 Å), the Mn^2+^ hydration spheres were not completely revealed and we could therefore not determine water-mediated contact sites of the metal ions in every case. One metal ion at G45 (from the first bp of the variable arm) seems to reinforce the interaction of that residue with U9 of the AD-linker (Figure 8C). While in general our results argue that divalent metal ions are not crucial for the stabilization of the tRNA^Sec^ structure, weak or incomplete metal ion binding sites in tRNA^Sec^ may be complemented by and thus serve as latching points for interacting proteins as seen, e.g, in certain metal ion-dependent RNases [58].

### Summary and conclusions

We elaborated a scheme for the fast, simple and reproducible production and purification of RNA molecules suitable for structural studies. The approach enables the combinatorial production of RNA variants, for example in order to screen for well diffracting crystal forms. This strategy allowed us to identify a variant of mouse tRNA^Sec^ that produced crystals suitable for high-resolution structure analysis.

We have determined a crystal structure of mouse tRNA^Sec^ at 2.0 Å resolution, which globally resembles structures of the closely related human tRNA^Sec^ determined at lower resolution alone and in complex with SecS. Compared to these previous structures, our work highlighted detailed aspects not seen before such as domain flexibility and the conformational spectrum of the anticodon stem-loop. We suggest that the molecule's flexibility provides an important indirect readout mechanism for interacting proteins and that tRNA^Sec^ can structurally adapt to the many different protein partners of the molecule. Additional co-crystal structures of tRNA^Sec^ with other proteins are required to fully explore the adaptability of the RNA. Only one of two different anticodon loop conformations we observed appears to be compatible with decoding on the ribosome and this form seems to be favored upon 2′-O-methylation of U34. We also were able to define in detail the hydration of tRNA^Sec^ and have analyzed its divalent metal ion binding potential. While water molecules are clearly important for the structural integrity, metal ions appear to be dispensable for stable tRNA^Sec^ folding.

## Materials and Methods

### Cloning of tRNA^Sec^, DNA template production and *in vitro* transcription

A DNA fragment containing a T7 RNA polymerase promoter, a sequence encoding mouse tRNA^Sec^ and restriction sites was PCR-assembled from DNA primers designed with the Assembly PCR Oligo Maker program [59]. The PCR product was cloned into the pUC19 plasmid by standard techniques. The sequence of the insert was verified by DNA sequencing.

The DNA template for *in vitro* transcription was generated by a two-step PCR (25 and 35 cycles) using a forward primer complementary to the T7 promoter and a reverse primer complementary to the 3′-end of the insert. The reverse primer contained two 2′-O-methylated nucleotides at the 5′ end, which suppress non-templated nucleotide addition by T7 RNA polymerase at the end of the coding region [28], [29]. The amplified DNA was extracted with phenol/chloroform/isoamylalcohol and precipitated by isopropanol. The pellet was washed twice with 70% EtOH, dried and dissolved in water.

RNAs were synthesized *in vitro* using T7 RNA polymerase as described earlier [60] with modifications. A PCR-amplified DNA fragment was used as a template at 10–15 µg per ml of transcription reaction. Preparative *in vitro* transcription (10 ml reaction) was done at 37°C for 3 h and the DNA template was subsequently digested with RNase-free RQ1 DNase. Transcribed RNA was analyzed by denaturing PAGE (15% acrylamide, 8 M urea) and visualized by staining with 0.01% methylene blue. The transcription mixture was stored frozen at −20°C until RNA purification.

### Non-denaturating purification of tRNA^Sec^

The RNA transcript was purified by anion exchange chromatography using a Mono Q 5/50 GL column (GE Healthcare). The column was equilibrated with buffer A (0.1 M Tris-HCl, pH 6.9, 0.4 M sodium acetate, 0.2 mM EDTA) and the sample was eluted with a linear gradient to buffer B (0.1 M Tris-HCl, pH 7.3, 1.5 M sodium acetate, 0.2 mM EDTA). Pooled peak fractions were concentrated by ultrafiltration using a 10,000 MWCO membrane (Millipore) and applied on a Superdex 75 gel filtration column (GE Healthcare) equilibrated with 10 mM HEPES-NaOH, pH 7.5, 50 mM NaCl. Fractions of tRNA^Sec^ were pooled, concentrated to 8 mg/ml, frozen in liquid nitrogen and stored at −80°C.

### Site-directed mutagenesis

Site-directed mutagenesis was performed according to the QuikChange™ strategy (Stratagene). Forward primer for substitution of the variable loop with a kissing loop: 5′-GCTTCAAACCTGTAGCTGTCAGGCGCGCAGACAGAGTGGTTCAATTCC-3′; reverse primer: 5′-GGAATTGAACCACTCTGTCTGCGCGCCTGACAGCTACAGGTTTGAAGC-3′. Forward primer for replacement of the variable loop by a UUCG tetraloop: 5′-GCTTCAAACCTGTAGCTGTCTTCGGACAGAGTGGTTCAATTCC-3′; reverse primer: 5′-GGAATTGAACCACTCTGTCCGAAGACAGCTACAGGTTTGAAGC-3′.

### Multi-angle static light scattering

Light scattering analysis was conducted at the laboratories of Wyatt Technology Europe GmbH. 5 µl of purified tRNA^Sec^ (8 mg/ml) were chromatographed on a Superdex 200 10/300 GL gel filtration column (GE Healthcare) in 20 mM HEPES-NaOH, pH 7.5, 200 mM NaCl at a flow rate of 0.5 ml/min using an Agilent 1200 HPLC system (Agilent Technologies). The elution was monitored *via* an Optilab rEX refractive index detector (Wyatt Technology), a DAWN Heleos II 18-angle light scattering detector (Wyatt Technology) and an Agilent 1200 variable wavelength detector (Agilent Technologies).

### Crystallographic procedures

For crystallization, 1 µl of ^ΔGCCA^tRNA^Sec^ (8 mg/ml) was mixed with an equal volume of reservoir solution (0.1 M MES, pH 5.2, 10 mM magnesium acetate, 2.0 M ammonium sulfate) in a 24-well CrysChem plate (Hampton Research). Crystals were grown by sitting-drop vapor diffusion at 20°C. They appeared within a few days and continued to grow for about one month. Micro-seeding was used to improve the initially very small crystals.

For diffraction data collection, crystals were transferred into cryo-protecting buffer (0.1 M MES, pH 5.2, 10 mM magnesium acetate, 2.0 M ammonium sulfate, 20% glycerol) and shock-frozen in liquid nitrogen. For Mn^2+^-binding studies, a crystal was soaked for 2 hours in a similar buffer, in which magnesium acetate was substituted with 100 mM manganese sulfate and 10 mM ammonium acetate, and shock-frozen in liquid nitrogen. Diffraction data for native and derivative crystals were collected at beamline 14.2 of BESSY (Berlin, Germany). Datasets were processed using the HKL package [61].

The structure was solved by molecular replacement with MOLREP [62] using fragments of human tRNA^Sec^ [24] as search models to locate molecule A (fragments used: nts 1–8 and 48–72 [acceptor/T-arms]; nts 9–45 [AD-linker/D-loop/anticodon arm]; nts 46–47l [variable arm]). Molecule B was subsequently located using the preliminary molecule A structure as the search model. The structure was completed by manual model building with COOT [63] and automatic refinement with Refmac5 [64]. Water molecules were automatically added with ARP/wARP [65] and the water structure was checked and completed manually. During all stages of refinement, a randomly selected set of 5% of the reflections was used for cross-validation.

Structure coordinates and diffraction data were deposited with the Protein Data Bank (http://www.pdb.org) under accession code 3RG5.

## References

[1] Allmang C, Krol A (2006). Selenoprotein synthesis: UGA does not end the story.. Biochimie.

[2] Ohama T, Yang DC, Hatfield DL (1994). Selenocysteine tRNA and serine tRNA are aminoacylated by the same synthetase, but may manifest different identities with respect to the long extra arm.. Arch Biochem Biophys.

[3] Forchhammer K, Böck A (1991). Selenocysteine synthase from Escherichia coli. Analysis of the reaction sequence.. J Biol Chem.

[4] Ehrenreich A, Forchhammer K, Tormay P, Veprek B, Böck A (1992). Selenoprotein synthesis in E. coli. Purification and characterisation of the enzyme catalysing selenium activation.. Eur J Biochem.

[5] Carlson BA, Xu XM, Kryukov GV, Rao M, Berry MJ (2004). Identification and characterization of phosphoseryl-tRNA[Ser]Sec kinase.. Proc Natl Acad Sci U S A.

[6] Araiso Y, Sherrer RL, Ishitani R, Ho JM, Söll D (2009). Structure of a tRNA-dependent kinase essential for selenocysteine decoding.. Proc Natl Acad Sci USA.

[7] Kaiser JT, Gromadski K, Rother M, Engelhardt H, Rodnina MV (2005). Structural and functional investigation of a putative archaeal selenocysteine synthase.. Biochemistry.

[8] Ganichkin OM, Xu XM, Carlson BA, Mix H, Hatfield DL (2008). Structure and catalytic mechanism of eukaryotic selenocysteine synthase.. J Biol Chem.

[9] Xu XM, Carlson BA, Mix H, Zhang Y, Saira K (2007). Biosynthesis of selenocysteine on its tRNA in eukaryotes.. PLoS Biol.

[10] Yuan J, Palioura S, Salazar JC, Su D, O'Donoghue P (2006). RNA-dependent conversion of phosphoserine forms selenocysteine in eukaryotes and archaea.. Proc Natl Acad Sci U S A.

[11] Araiso Y, Palioura S, Ishitani R, Sherrer RL, O'Donoghue P (2008). Structural insights into RNA-dependent eukaryal and archaeal selenocysteine formation.. Nucleic Acids Res.

[12] Stock T, Selzer M, Rother M (2010). In vivo requirement of selenophosphate for selenoprotein synthesis in archaea.. Mol Microbiol.

[13] Heider J, Baron C, Böck A (1992). Coding from a distance: dissection of the mRNA determinants required for the incorporation of selenocysteine into protein.. EMBO J.

[14] Walczak R, Westhof E, Carbon P, Krol A (1996). A novel RNA structural motif in the selenocysteine insertion element of eukaryotic selenoprotein mRNAs.. RNA.

[15] Forchhammer K, Leinfelder W, Böck A (1989). Identification of a novel translation factor necessary for the incorporation of selenocysteine into protein.. Nature.

[16] Copeland PR, Fletcher JE, Carlson BA, Hatfield DL, Driscoll DM (2000). A novel RNA binding protein, SBP2, is required for the translation of mammalian selenoprotein mRNAs.. EMBO J.

[17] Fagegaltier D, Hubert N, Yamada K, Mizutani T, Carbon P (2000). Characterization of mSelB, a novel mammalian elongation factor for selenoprotein translation.. EMBO J.

[18] Caban K, Copeland PR (2006). Size matters: a view of selenocysteine incorporation from the ribosome.. Cell Mol Life Sci.

[19] Leibundgut M, Frick C, Thanbichler M, Böck A, Ban N (2005). Selenocysteine tRNA-specific elongation factor SelB is a structural chimaera of elongation and initiation factors.. EMBO J.

[20] Small-Howard A, Morozova N, Stoytcheva Z, Forry EP, Mansell JB (2006). Supramolecular complexes mediate selenocysteine incorporation in vivo.. Molecular and Cellular Biology.

[21] Hubert N, Sturchler C, Westhof E, Carbon P, Krol A (1998). The 9/4 secondary structure of eukaryotic selenocysteine tRNA: more pieces of evidence.. RNA.

[22] Baron C, Westhof E, Böck A, Giege R (1993). Solution structure of selenocysteine-inserting tRNA(Sec) from Escherichia coli. Comparison with canonical tRNA(Ser).. J Mol Biol.

[23] Ioudovitch A, Steinberg SV (1998). Modeling the tertiary interactions in the eukaryotic selenocysteine tRNA.. RNA.

[24] Itoh Y, Chiba S, Sekine S, Yokoyama S (2009). Crystal structure of human selenocysteine tRNA.. Nucleic Acids Res.

[25] Hatfield DL, Gladyshev VN (2002). How selenium has altered our understanding of the genetic code.. Mol Cell Biol.

[26] Motorin Y, Helm M (2010). tRNA stabilization by modified nucleotides.. Biochemistry.

[27] Palioura S, Sherrer RL, Steitz TA, Soll D, Simonovic M (2009). The human SepSecS-tRNASec complex reveals the mechanism of selenocysteine formation.. Science.

[28] Kao C, Zheng M, Rudisser S (1999). A simple and efficient method to reduce nontemplated nucleotide addition at the 3 terminus of RNAs transcribed by T7 RNA polymerase.. RNA.

[29] Sherlin LD, Bullock TL, Nissan TA, Perona JJ, Lariviere FJ (2001). Chemical and enzymatic synthesis of tRNAs for high-throughput crystallization.. RNA.

[30] Lodmell JS, Ehresmann C, Ehresmann B, Marquet R (2001). Structure and dimerization of HIV-1 kissing loop aptamers.. J Mol Biol.

[31] Byrne RT, Konevega AL, Rodnina MV, Antson AA (2010). The crystal structure of unmodified tRNAPhe from Escherichia coli.. Nucleic Acids Res.

[32] Chiba S, Itoh Y, Sekine S, Yokoyama S (2010). Structural basis for the major role of O-phosphoseryl-tRNA kinase in the UGA-specific encoding of selenocysteine.. Mol Cell.

[33] Sherrer RL, Araiso Y, Aldag C, Ishitani R, Ho JM (2011). C-terminal domain of archaeal O-phosphoseryl-tRNA kinase displays large-scale motion to bind the 7-bp D-stem of archaeal tRNASec.. Nucleic Acids Research.

[34] Wahl MC, Rao ST, Sundaralingam M (1996). The structure of r(UUCGCG) has a 5′-UU-overhang exhibiting Hoogsteen-like trans U.U base pairs.. Nat Struct Biol.

[35] Wahl MC, Sundaralingam M (1997). C-H…O hydrogen bonding in biology.. Trends Biochem Sci.

[36] Holbrook SR, Cheong C, Tinoco I, Kim SH (1991). Crystal structure of an RNA double helix incorporating a track of non-Watson-Crick base pairs.. Nature.

[37] Cabello-Villegas J, Winkler ME, Nikonowicz EP (2002). Solution conformations of unmodified and A(37)N(6)-dimethylallyl modified anticodon stem-loops of Escherichia coli tRNA(Phe).. J Mol Biol.

[38] Heus HA, Pardi A (1991). Structural features that give rise to the unusual stability of RNA hairpins containing GNRA tetraloops.. Science.

[39] Cheong C, Varani G, Tinoco I (1990). Solution structure of an unusually stable RNA hairpin, 5′GGAC(UUCG)GUCC.. Nature.

[40] Correll CC, Wool IG, Munishkin A (1999). The two faces of the Escherichia coli 23 S rRNA sarcin/ricin domain: the structure at 1.11 A resolution.. J Mol Biol.

[41] Jucker FM, Heus HA, Yip PF, Moors EH, Pardi A (1996). A network of heterogeneous hydrogen bonds in GNRA tetraloops.. J Mol Biol.

[42] Alexander RW, Eargle J, Luthey-Schulten Z (2010). Experimental and computational determination of tRNA dynamics.. FEBS Lett.

[43] Robertus JD, Ladner JE, Finch JT, Rhodes D, Brown RS (1974). Structure of yeast phenylalanine tRNA at 3 A resolution.. Nature.

[44] Steitz TA (2008). A structural understanding of the dynamic ribosome machine.. Nat Rev Mol Cell Biol.

[45] Vendeix FA, Dziergowska A, Gustilo EM, Graham WD, Sproat B (2008). Anticodon domain modifications contribute order to tRNA for ribosome-mediated codon binding.. Biochemistry.

[46] Diamond AM, Choi IS, Crain PF, Hashizume T, Pomerantz SC (1993). Dietary selenium affects methylation of the wobble nucleoside in the anticodon of selenocysteine tRNA([Ser]Sec).. J Biol Chem.

[47] Warner GJ, Berry MJ, Moustafa ME, Carlson BA, Hatfield DL (2000). Inhibition of selenoprotein synthesis by selenocysteine tRNA([Ser]Sec) lacking isopentenyladenosine.. J Biol Chem.

[48] Joachimiak A, Haran TE, Sigler PB (1994). Mutagenesis supports water mediated recognition in the trp repressor-operator system.. EMBO J.

[49] Walter NG (2007). Ribozyme catalysis revisited: Is water involved?. Mol Cell.

[50] Westhof E (1988). Water - an Integral-Part of Nucleic-Acid Structure.. Annu Rev Biophys Biophys Chem.

[51] Kennard O, Cruse WB, Nachman J, Prange T, Shakked Z (1986). Ordered water structure in an A-DNA octamer at 1.7 A resolution.. J Biomol Struct Dyn.

[52] Shi HJ, Moore PB (2000). The crystal structure of yeast phenylalanine tRNA at 1.93 angstrom resolution: A classic structure revisited.. RNA.

[53] Biswas R, Wahl MC, Ban C, Sundaralingam M (1997). Crystal structure of an alternating octamer r(GUAUGUA)dC with adjacent G×U wobble pairs.. J Mol Biol.

[54] Mueller U, Schubel H, Sprinzl M, Heinemann U (1999). Crystal structure of acceptor stem of tRNA(Ala) from Escherichia coli shows unique G.U wobble base pair at 1.16 A resolution.. RNA.

[55] Cate JH, Doudna JA (1996). Metal-binding sites in the major groove of a large ribozyme domain.. Structure.

[56] Gueron M, Leroy JL (1982). Significance and mechanism of divalent ion binding to transfer RNA.. Biophys J.

[57] Ennifar E, Yusupov M, Walter P, Marquet R, Ehresmann B (1999). The crystal structure of the dimerization initiation site of genomic HIV-1 RNA reveals an extended duplex with two adenine bulges.. Structure.

[58] Song JJ, Smith SK, Hannon GJ, Joshua-Tor L (2004). Crystal structure of Argonaute and its implications for RISC slicer activity.. Science.

[59] Rydzanicz R, Zhao XS, Johnson PE (2005). Assembly PCR oligo maker: a tool for designing oligodeoxynucleotides for constructing long DNA molecules for RNA production.. Nucleic Acids Res.

[60] Pokrovskaya ID, Gurevich VV (1994). In vitro transcription: preparative RNA yields in analytical scale reactions.. Anal Biochem.

[61] Otwinowski Z, Minor W (1997). Processing of X-ray diffraction data collected in oscillation mode.. Macromol Crystallogr.

[62] Vagin A, Teplyakov A Molecular replacement with MOLREP.. Acta Crystallogr D.

[63] Emsley P, Lohkamp B, Scott WG, Cowtan K (2010). Features and development of Coot.. Acta Crystallogr D.

[64] Murshudov GN, Vagin AA, Dodson EJ (1997). Refinement of macromolecular structures by the maximum-likelihood method.. Acta Crystallogr D.

[65] Perrakis A, Morris R, Lamzin VS (1999). Automated protein model building combined with iterative structure refinement.. Nat Struct Biol.

